# Acute kidney injury associated with increased costs in the neonatal intensive care unit: analysis of Pediatric Health Information System database

**DOI:** 10.1038/s41372-024-02193-x

**Published:** 2024-12-05

**Authors:** Heidi J. Steflik, David T. Selewski, Corinne Corrigan, Daniel L. Brinton

**Affiliations:** 1 Department of Pediatrics, Medical University of South Carolina, Charleston, SC USA; 2 Premier Inc., Charlotte, NC USA; 3 Department of Healthcare Leadership and Management, Medical University of South Carolina, Charleston, SC USA

**Keywords:** Epidemiology, Kidney diseases

## Abstract

**Objective:**

Compare neonatal intensive care unit hospitalization costs between neonates with and without AKI; identify predictors of AKI-associated costs. We hypothesized neonates with AKI would amass more costs than those without AKI.

**Study design:**

Retrospective, multicenter cohort study of surviving neonates cared for 2015–2021 in Pediatric Health Information System database. The primary outcome was estimated hospitalization costs.

**Results:**

Data from 304,725 neonates were evaluated, 8774 (3%) with AKI and 295,951 (97%) without AKI. Neonates with AKI had $58,807 greater adjusted costs than those without AKI. AKI-associated costs were most strongly driven by Feudtner Pediatric Complex Chronic Conditions Classifications (cardiovascaular, congenital/genetic, gastrointestinal, medical technology) and gestational age. Adjusted costs decreased with increasing gestational age, regardless of AKI status.

**Conclusions:**

AKI is independently associated with increased hospital costs. Knowledge of these drivers can help in identifying high-value practices for cost mitigation strategies.

## Introduction

Neonatal acute kidney injury (AKI), once thought to be an infrequent complication with few short- or long-term consequences, is now known to occur commonly in the neonatal intensive care unit (NICU) and independently impact outcomes during the NICU stay and later in life. In the seminal, multicenter Assessment of Worldwide Acute Kidney Injury Epidemiology in Neonates (AWAKEN) study, investigators found AKI was an independent risk factor for both mortality and longer hospital stay [1]. The impacts of AKI are likely present long after NICU discharge as well. The Follow-Up of AKI in Neonates during Childhood Years (FANCY) study investigators found that former very low birth weight (VLBW, i.e., <1500 g at birth) preterm infants who experienced AKI in the NICU were more likely to have kidney dysfunction at 3–7 years of age than those without AKI [2].

The incidence of AKI in the NICU and these significant impacts suggest the economic burden of neonatal AKI could be substantial, but the financial consequences of neonatal AKI remain understudied. Studies in adults and pediatric populations demonstrate AKI is independently associated with prolonged hospitalizations and excessive hospitalization costs [3–8]. In neonates, our team recently conducted an exploratory, pilot project using the Children’s Hospital Association’s (CHA) Pediatric Health Information System (PHIS) database to ascertain the marginal estimated total cost of hospitalization in a sub-population of neonates at particularly high risk for AKI, those in the NICU with patent ductus arteriosus (PDA). We found among neonates with PDA, those who developed AKI had an adjusted $48,416 greater costs (95% CI: $43,804–$53,227) than those who did not develop AKI [9]. Specific predictors of costs in this analysis (all *p* < 0.01) included AKI, birthweight, ethnicity, race, length of hospitalization (LOH), and Feudtner Pediatric Complex Chronic Conditions Classification (CCC) status. To our knowledge, this is the first publication to examine costs of AKI in the NICU, but understanding the costs in a broader NICU cohort is necessary and the logical next step. Only when the costs associated with neonatal AKI are established can we begin to identify high-value practices for prevention of neonatal AKI and cost mitigation strategies.

We sought to begin to fill this knowledge gap and specifically aimed to (1) determine and compare estimated costs of hospitalization between neonates in the NICU who did and did not develop AKI and (2) identify predictors of AKI-associated costs. We hypothesized neonates with AKI would amass significantly more costs than those without AKI, and these increased costs would be driven by premature birth, LOH, and renal replacement therapy (RRT) receipt.

## Materials/subjects and methods

### Data source and study population

We conducted a retrospective cohort study of neonates cared for in the NICU using the CHA PHIS database. Comprising 49 children’s hospitals in the United States, PHIS is a comparative, pediatric administrative database that includes clinical and resource utilization data from inpatient, ambulatory surgery, emergency department, and observational unit patient encounters. Surviving neonates cared for in a NICU in a CHA PHIS center (indicated by the presence of a database ‘NICU flag’) between 01/01/2015 and 12/31/2021 were included, and those who had duplicate entries or in whom the discharge year was undocumented were excluded.

### Data collection

For each subject, de-identified encrypted data including baseline demographic and clinical characteristics, CHA PHIS center, all International Classification of Disease, Ninth and Tenth Revision, Clinical Modification (ICD-9/ICD-10-CM) diagnosis codes, and all ICD-9 and ICD-10 procedure codes from the NICU encounter as well as LOH and survival were collected.

Cost-to-charge ratio (CCR)-based adjusted total costs, expressed in United States dollars, were also collected for each subject. Within the PHIS database, estimated costs for each patient encounter is determined by multiplying the abstract-based charges by a CCR for that encounter. The CCR is based on the center and discharge year and determined by the official cost reports submitted to the Centers for Medicare and Medicaid Services annually. If the cost report is unavailable, each center is asked to provide the CCR using a provided formula.

To quantify degree of illness and comorbid burden among our study subjects, we used the CCC system, version 2.0 [10]. Feudtner and colleagues define CCC’s as the following: “any medical condition that can reasonably be expected to last at least 12 months (unless death intervenes) and to involve either several different organ systems or 1 organ system severely enough to require specialty pediatric care and probably some period of hospitalization in a tertiary care center” [11]. Each subject’s ICD-10 diagnosis and procedure codes are used to determine if a single or multiple Feudtner CCC’s were present and, if present, to categorize them into one of the following potential CCC categories: cardiovascular, respiratory, neuromuscular, renal, gastrointestinal, hematologic or immunologic, metabolic, other congenital or genetic, malignancy, and premature or neonatal complex chronic conditions (Supplementary Table 1). RRT receipt was determined by presence or absence of ICD-10 procedure codes for RRT (5A1D70Z, 5A1D80Z, 5A1D90z).

### Statistical analysis

The presence or absence of AKI defined the two comparison groups and was determined by the presence or absence of an ICD-10-CM diagnosis code for AKI (N17.0, N17.1, N17.2, N17.8, N17.9, N19, N99.0, R34) [12]. Race was designated as Asian/Pacific Islander/American Indian, Black, White, Other, and Unknown. Gestational age (in completed weeks) was grouped as: 22–26, 27–30, 31–34, 35–37, and ≥38 weeks of gestation, and birthweight (in grams) was grouped as: <500, 500–999, 1000–1499, 1500–2499, and ≥2500 g in accordance with prior studies, but with smaller groups to allow for improved discrimination [1, 13]. Baseline and demographic data were compared between those with and without AKI using Wilcoxon–Mann Whitney and Chi-squared tests. Continuous variables are presented as medians [interquartile ranges] for non-normally distributed variables and mean ± standard deviation for normally distributed variables. Categorical variables were presented as frequency (proportions).

The primary outcome was estimated hospitalization costs, expressed as estimated, adjusted marginal total costs of index NICU hospitalization. The estimated adjusted, marginal total cost of hospitalization was estimated using a gamma-distributed log-transformed link function generalized linear model. Costs were inflation-adjusted to March 2022 United States dollars using the Consumer Price Index for inpatient hospital services [14]. Notably, the Feudtner ‘Neonatal’ CCC includes ICD-10-CM codes for small for gestational age as well as for disorders of newborn related to low birth weight and thus was eliminated from multivariable modeling to avoid multicollinearity. Additionally, we detected collinearity between birthweight and gestational age. Thus, to avoid multicollinearity, we utilized gestational age (rather than birthweight) for our analyses. The final model was adjusted for factors identified a priori that might bias cost comparisons including race, ethnicity, sex, gestational age, Feudtner CCC, and RRT. Multiple imputation by chained equations was employed for missing data, specifically gestational age, as we believe the missing data mechanism to be missing at random and the amount of missing data was low. Additionally, we report adjusted total costs by the pre-defined gestational age groups among neonates with and without AKI with associated 95% confidence intervals.

Among neonates with AKI, key predictors of AKI-associated costs were identified using a logistic regression model, modeling the outcome of development of AKI. Backwards model fitting was used, entering all potential predictors into the model and removing one at a time until model parsimony was reached. Final model fit was assessed using the modified, Hosmer-Lemeshow goodness of fit test for large datasets [15].

Given LOH is expected to be longer in more prematurely born neonates, we examined trends in LOH by gestational age among neonates who experienced AKI using a Jonckheere-Terpstra test [16–18].

Given 19% of those with AKI belonged to the 22–26 weeks’, a *post-hoc* subgroup analysis was performed examining the cost differences (and key drivers of cost) between those with and without AKI among subjects who were born between 22 and 26 weeks’ gestation using the previously-detailed analytical approach.

All analyses were performed using SAS version 9.4 (SAS Institute, Cary NC), and a significance was determined to be at the $\alpha$=0.05 level a priori. Methods and results are reported in accordance with the Strengthening the Reporting of Observational Studies in Epidemiology (STROBE) guidelines [19].

This study was approved by the Medical University of South Carolina Institutional Review Board. A waiver of informed consent was granted given the retrospective data collection process and the anonymity of protected health information (de-identified data). This study was performed in accordance with the Declaration of Helsinki.

## Results

**Fig. 1:**
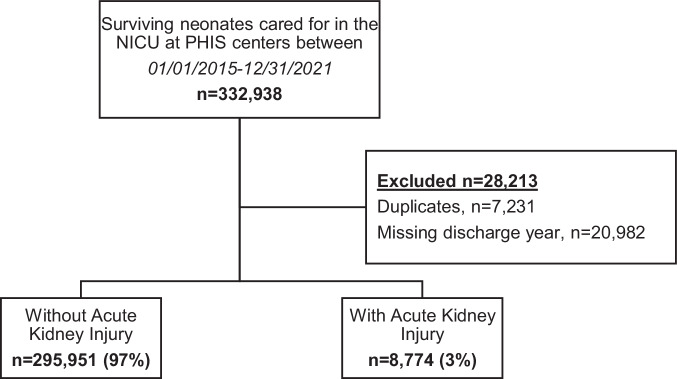
Flow diagram of study enrollment.

Data from 304,725 neonates, 8774 (3%) with AKI and 295,951 (97%) without AKI, were included (Fig. 1). Significant differences were found in every demographic/baseline characteristic between those with and without AKI; neonates with AKI were primarily non-Hispanic white males and most frequently ≥2500 g and ≥38 weeks’ gestational age at birth (Table 1). A higher proportion of neonates developed AKI from 2019 to 2021. Neonates with AKI comprised a higher proportion of each Feudtner CCC category except malignancy.

**Table 1:** Demographic and baseline characteristics of neonates with and without AKI.

Demographics and characteristic		Acute kidney injury	
		No (*n* = 295,951)	Yes (*n* = 8774)
Sex	Male	159,097 (53.8)	5257 (59.9)
	Female	136,620 (46.1)	3505 (40.0)
Race	Asian/Pacific Islander/ American Indian	13,879 (4.7)	383 (4.4)
	Black	52,046 (17.6)	1870 (21.3)
	White	160,661 (54.3)	4657 (53.1)
	Other	37,236 (12.6)	1176 (13.4)
	Unknown	32,129 (10.9)	688 (7.8)
Ethnicity	Hispanic or Latino	45,503 (15.4)	1838 (20.9)
	Not Hispanic or Latino	217,324 (73.4)	6315 (72.0)
	Unknown	33,124 (11.2)	621 (7.1)
Birthweight	<500 g	1000 (0.3)	140 (1.6)
	≥500–<1000 g	13,403 (4.5)	1894 (21.6)
	≥1000–<1500 g	17,893 (6.0)	566 (6.5)
	≥1500–<2500 g	67,768 (22.9)	1238 (14.1)
	≥2500 g	161,976 (54.7)	3955 (45.1)
	Missing	33,911 (11.5)	981 (11.2)
Gestational age	22–26 weeks’	9991 (3.4)	1682 (19.2)
	27–30 weeks’	17,278 (5.8)	669 (7.6)
	31–34 weeks	45,878 (15.5)	764 (8.7)
	35–37 weeks’	60,955 (20.6)	1433 (16.3)
	≥38 weeks’	94,963 (32.1)	2475 (28.2)
	Missing	66,886 (22.6)	1751 (20.0)
Discharge year	2015	43,370 (14.7)	931 (10.6)
	2016	47,727 (16.1)	1227 (14.0)
	2017	51,324 (17.3)	1398 (15.9)
	2018	45,749 (15.5)	1293 (14.7)
	2019	44,308 (15.0)	1483 (16.9)
	2020	35,445 (12.0)	1378 (15.7)
	2021	28,028 (9.5)	1064 (12.1)
Renal replacement therapy	Yes	167 (0.1)	243 (2.8)
Feudtner^a^ Pediatric Complex Chronic Conditions (CCC) Classifications	Any CCC?	144,471 (48.8)	8261 (94.2)
	# of CCC’s	0.9 ± 1.4	2.9 ± 1.9
	Cardiovascular	40,942 (13.8)	3839 (43.8)
	Congenital or Genetic	20,450 (6.9)	1278 (14.6)
	Gastrointestinal	29,877 (10.1)	2960 (33.7)
	Hematologic or Immunologic	7061 (2.4)	704 (8.0)
	Malignancy	10,724 (3.6)	213 (2.4)
	Metabolic	14,858 (5.0)	1293 (14.7)
	Neonatal	70,608 (23.9)	6187 (70.5)
	Neurologic or Neuromuscular	21,110 (7.1)	1594 (18.2)
	Renal	16,362 (5.5)	2498 (28.5)
	Respiratory	16,040 (5.4)	1383 (15.8)
	Medical Technology (i.e., device dependency)	27,051 (9.1)	3298 (37.6)
	Transplantation	5510 (1.9)	217 (2.5)

Continuous variables presented as mean ± standard deviation; Categorical variables presented as counts (percentages). All comparisons were statistically significant (*p* < 0.0001) due to large group sizes.

^a^Feudtner et al. BMC Pediatrics 2014, 14:199.

**Fig. 2:**
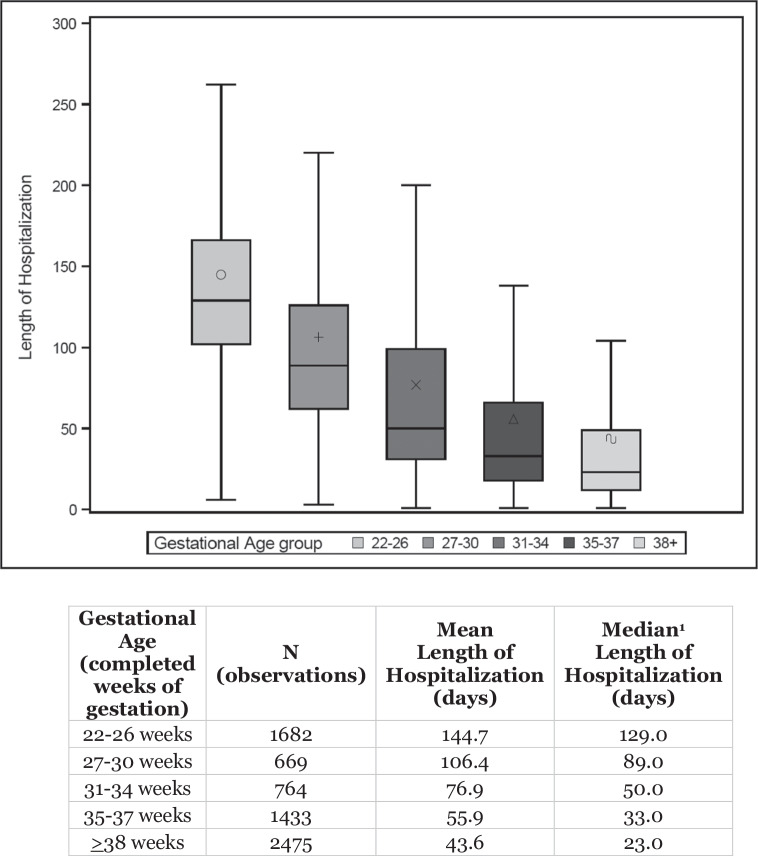
Length of hospitalization by gestational age group among neonates with acute kidney injury. Box-and-whiskers plot illustrating length of hospitalization among the various gestational age groups. The Box is defined by the 25^th^ and 75^th^ percentiles. Within the Box, the mean length of hospitalization is indicated by the notation (open circle, cross, X, triangle, curved line, respectively) and the median length of hospitalization is indicated by the horizontal bold bar. The whiskers indicated the minimum and maximum observations. ^1^Significant for non-linear trend (*p* < 0.0001) using the Jonckheere-Terpstra test.

Among neonates with AKI, when examining LOH as it relates to GA, we detected a significant trend: as gestational age increases, LOH decreases (*p* < 0.0001; Fig. 2). We detected a similar, significant trend among neonates without AKI (Supplemental Figure). When comparing LOH between those with and without AKI, those with AKI experienced significantly longer hospital stays (no AKI: mean 22.6 ± 38.4 days vs. AKI: 78.6 ± 82.8 days; *p* < 0.0001).

Neonates with AKI had, on average, $58,807 greater costs than those without AKI after adjusting for predictors (*p* < 0.0001; Table 2). Significant predictors of costs included race, ethnicity, sex, gestational age, Feudtner CCC’s, and RRT (all *p* < 0.01). We found similar results when multiple imputation methodologies for gestational age missingness were used; neonates with AKI had, on average, $53,634 greater costs than those without AKI after adjusting for predictors.

**Table 2:** Comparison of adjusted cost of hospitalization between neonates with and without AKI.

Models	No AKI (*n* = 295,951)	AKI (*n* = 8,774)	*p*-value
Complete case analysis^a^	$60,705 ($60,473-$60,937)	$119,512 ($116,935-$122,147)	<0.0001
Multiple imputation analysis^a,b^	$55,649 ($55,453-$55,846)	$109,283 ($107,066-$111,546)	<0.0001

^a^All predictors were significant in the model and adjusted for: race, ethnicity, sex, gestational age, Feudtner complex chronic conditions classifications, and renal replacement therapy.

^b^Multiple imputation for gestational age employed in this model.

Among neonates with AKI (*n* = 8774), the top 5 key drivers of AKI-associated costs included the presence of four Feudtner CCCs (cardiovascular, congenital or genetic, gastrointestinal, medical technology) and gestational age (Supplementary Table 2). When examining costs by pre-specified gestational age subgroups, adjusted costs decreased with increasing gestational age in neonates with and without AKI (Table 3).

**Table 3:** Adjusted costs by gestational age among neonates with and without acute kidney injury.

Gestational age (completed weeks of gestation)	Neonates without AKI			Neonates with AKI		
	Adjusted Cost^a^	95% Confidence Interval		Adjusted Cost^b^	95% Confidence Interval	
22–26 weeks	$267,941	$263,153	$272,816	$622,252	$600,140	$645,178
27–30 weeks	$209,500	$206,553	$212,490	$467,431	$442,501	$493,764
31–34 weeks	$87,858	$87,084	$88,638	$272,735	$258,715	$287,515
35–37 weeks	$43,060	$42,726	$43,396	$206,398	$198,023	$215,127
≥38 weeks	$34,234	$34,033	$34,437	$191,251	$185,668	$197,003

^a,b^Significant for non-linear trend (*p* < 0.0001) using the Jonckheere-Terpstra test.

In the *post-hoc* subgroup analysis examining costs and drivers among neonates born 22–26 weeks of gestation, our findings were similar to those in the main cohort analysis; Neonates born 22–26 weeks of gestation who developed AKI experienced greater costs than those without AKI after adjusting for predictors (no AKI: $401,039 (95% CI $394,623–$407,560) vs. AKI: $544,251 (95% CI $523,067–$566,292)). The 3 strongest drivers of cost (rank-ordered) in this subgroup differed from those in the overall cohort and were CCC-Respiratory, CCC-GI, and AKI.

## Discussion

While an increasing number of studies suggest AKI is frequent and imparts significant morbidity and increased risk for mortality in neonates cared for in the NICU, the economic burden associated with neonatal AKI remains largely unknown. In this singular examination of costs associated with AKI in a broad cohort of surviving neonates cared for in the NICU, our findings support our primary hypotheses. In this cohort, AKI development is associated with significant increases in hospitalization costs after controlling for differences that suggest those with AKI are simply a sicker population (including race, ethnicity, sex, gestational age, Feudtner CCC, and RRT). Neonates who developed AKI had, on average, $58,807 greater costs than those who did not develop AKI. Furthermore, those greater costs are most strongly driven by degree of illness, here quantified by Feudtner CCC status, and gestational age, which is strongly, inversely associated with LOH. However, contrary to our hypothesis, RRT receipt was not a key driver of AKI-associated costs, likely due to the low incidence of RRT receipt in our cohort (<0.15%).

Neonatal AKI is common, affecting an estimated 30% of neonates in the NICU, and is associated with both short- and long-term morbidity as well as mortality [1]. Despite this high prevalence, studies to improve our understanding of the economic impacts of AKI are limited, and few publications are available examining the costs associated with AKI development, particularly in neonates. In our recent exploratory pilot project, our group found AKI development was independently associated with significant increases in hospitalization costs in neonates with PDA in the NICU, a group at particularly high risk for AKI [20–22]. Though we found AKI to be associated with higher costs in this high risk NICU subpopulation, a deeper examination of the costs attributable to AKI in a broad NICU cohort was, until now, missing.

In pediatric and adult patients, AKI is independently associated with prolonged hospitalizations and excessive hospitalization costs [3–5]. In the NICU, however, hospitalizations costs are markedly variable based on gestational age [23–25]. With improving technology, increasing numbers of extremely premature and peri-viable neonates are surviving to hospital discharge, but the costs for care of these fragile neonates are high [26]. In all 18 studies included in a review published in 2014 assessing the cost of prematurity according to gestational age at birth, costs were inversely related to gestational age, and these findings are corroborated in subsequent publications [23, 25, 27, 28]. In the short-term (i.e., during the first year of life), mean costs for extremely premature neonates ranges from $12,910 to $297,627 [23, 27, 29]. However, when focusing solely on NICU costs, extremely preterm neonates account for only a small percentage of the total NICU costs, with total NICU expenditures skewed toward the care of moderate and late preterm infants [24, 25]. With these findings in mind, here we have examined cost in pre-specified gestational age-based subgroups; in both those with and without AKI, adjusted costs decreased with increasing gestational age. Similarly, median LOH decreased with increasing gestational age in both those with and without AKI. Significant differences in the populations studied, may, in part, explain the significantly lower costs attributable to AKI noted here compared to our previous findings in neonates with PDA and AKI which were noted in a much smaller cohort with significantly fewer older patients with shorter lengths of stay.(9). Additionally, modeling strategies employed in this analysis were selected, recognizing that LOH and costs are endogenous, i.e., LOH affects costs but costs also influence LOH. Using LOH as a predictor or covariate when modeling costs can result in spurious correlations and inaccurate estimates of the true relationships between the modeled predictors and the outcomes (here, total costs) [30].

Costs of other morbidities associated with prematurity have been examined [28, 31]. Russell et al. found mean hospital costs for preterm infants with common morbidities of prematurity including respiratory distress syndrome (RDS), bronchopulmonary dysplasia (BPD), intraventricular hemorrhage, and necrotizing enterocolitis (NEC) are four to seven times higher than costs in gestational-age equivalent healthy controls [28]. In this study using data from the 2001 Nationwide Inpatient Sample from Healthcare Cost and Utilization Project, the single costliest comorbidity of prematurity, expressed as average cost per discharge, was BPD with an average cost of $115,000. However, this comorbidity was only noted in 4.4% of analyzed cases. In contrast, the average cost for stays with RDS was less at $56,800, however 23.3% of infants studied were impacted. When examining direct costs for the initial NICU hospitalization and potentially preventable comorbidities of prematurity, Johnson et al. found costs were increased by $12,048 with the presence of brain injury, $15,400 with NEC, $31,565 with BPD, and $10,055 with late-onset sepsis after controlling for birthweight, gestational age, and sociodemographic characteristics [31]. Notably, costs associated with AKI were not examined in either of these important studies. Encouragingly, despite the high costs of care in some circumstances, studies confirm that neonatal intensive care is very cost-effective at $1000 per term infant per quality-adjusted life year (QALY) and $9100 per extremely premature neonatal survivor per QALY [25].

*Post-hoc* analysis of the 22–26 weeks of gestational age cohort was performed given this subgroup represents 19% of those with AKI. However, these results should be interpreted with caution as this subgroup represents merely 3.8% of the overall cohort. Notably, the scale of the cost differences between the overall cohort ($58,807) and this subgroup ($143,212) is remarkable. In the overall cohort, AKI increased costs 96.9%, however in this subgroup, AKI increased costs only 35.7%. Additionally key drivers of AKI-associated costs differed in this subgroup from the overall cohort suggesting the costs associated with AKI development in this subgroup may be distinct.

Establishing the costs associated with neonatal AKI is a critical first step before true cost-effectiveness studies can be undertaken. Only with these costs quantified can we begin to understand which of several potential high-value practices for prevention of neonatal AKI, such as systemic surveillance programs to monitor nephrotoxin exposure and creatinine for example, are most cost-effective. In the future, this groundwork may also help to identify the most cost-effective therapeutic options for this costly pathology.

A number of strengths and limitations require acknowledgement. Few studies have examined the costs associated with development of specific comorbidities in the NICU, and to our knowledge, none have examined the costs of AKI specifically in a population of neonates cared for in the NICU. As such, this data is the first of its kind, and studies designed to identify high-value practices and interventions for prevention of neonatal AKI and cost mitigation strategies can be performed. We utilized novel strategies to ascertain the costs associated with AKI and employed both multiple imputation analysis for missing gestational age data and examined cost differences by gestational-age based groups given the association between gestational age and LOH. Additionally, we used the Feudtner CCC system to quantify degree of illness in these analyses. This system has been used in prior, noteworthy publications, but we believe this is an important point of novelty for these analyses [32, 33]. Additionally the Feudtner CCC system allowed us to include, into the assessment of degree illness, comorbidities which occurred throughout the index hospitalization rather than datapoints from solely the first 12 hours of life, as is often the case in other scoring systems frequently used for this purpose in neonates. As our group found in our pilot study, though the PHIS database is an exceptional administrative database for cost estimation, clinical information including AKI incidence is limited due to the retrospective nature of the database. Here AKI is determined by the presence or absence of an ICD-10-CM diagnosis code for AKI, and multiple recent studies confirm AKI recognition in the NICU often remains quite low despite increasing study [34, 35]. As such, our AKI incidence (3%) is likely an underestimation of the true AKI incidence, though it is noteworthy that AKI incidence was slightly higher in the 2019–2021 groups than in the 2015–2018 groups. Our study excluded non-survivors which impacts anticipated AKI incidence but also improves the generalizability of our findings. Other limitations include the retrospective nature of this project and the non-inclusion of infant with and without AKI who did not survive. Additionally, though we utilized multiple imputation techniques to account for missingness of the gestational age variable, missingness of other data points was not addressed.

In this analysis of the economic burden of AKI in the NICU, AKI is independently associated with increased hospital costs. The key drivers of costs of hospitalization in neonates with AKI in the NICU included four Feudtner CCCs (cardiovascular, congenital or genetic, gastrointestinal, medical technology) and gestational age. Importantly, associations between gestational age and LOH were noted. The estimated hospitalizations costs decreased with increasing gestational age in those with and those without AKI. These findings add to a slowly growing body of literature informing the costs of care of critically ill neonates in the NICU and, in particular, costs of comorbidities associated with prematurity. Knowledge of these cost and their key drivers can help in identifying high-value practices and interventions that may provide cost mitigation and inform future studies.

## Supplementary information

Suplemental Figure

## Data Availability

Data analyzed in this study was provided by the Children’s Hospital Association and derived from the Pediatric Health Information System (PHIS) database. Per the data use agreement, we are unable to share these data. However, SAS code is available if requested.

## References

[1] Jetton JG, Boohaker LJ, Sethi SK, Wazir S, Rohatgi S, Soranno DE, et al. Incidence and outcomes of neonatal acute kidney injury (AWAKEN): a multicentre, multinational, observational cohort study. Lancet Child Adolesc Health. 2017;1:184–94. 10.1016/s2352-4642(17)30069-x. 29732396 10.1016/S2352-4642(17)30069-X PMC5933049

[2] Harer MW, Pope CF, Conaway MR, Charlton JR. Follow-up of Acute kidney injury in Neonates during Childhood Years (FANCY): a prospective cohort study. Pediatr Nephrol. 2017;32:1067–76. 10.1007/s00467-017-3603-x. 28255805 10.1007/s00467-017-3603-x

[3] Hobson C, Ozrazgat-Baslanti T, Kuxhausen A, Thottakkara P, Efron PA, Moore FA, et al. Cost and Mortality Associated With Postoperative Acute Kidney Injury. Ann Surg. 2015;261:1207–14. 10.1097/sla.0000000000000732. 24887982 10.1097/SLA.0000000000000732 PMC4247993

[4] Silver SA, Long J, Zheng Y, Chertow GM. Cost of Acute Kidney Injury in Hospitalized Patients. J Hosp Med. 2017;12:70–76. 10.12788/jhm.2683. 28182800 10.12788/jhm.2683

[5] McCormick M, Richardson T, Warady BA, Novelli EM, Kalpatthi R. Acute kidney injury in paediatric patients with sickle cell disease is associated with increased morbidity and resource utilization. Br J Haematol. 2020;189:559–65. 10.1111/bjh.16384. 32030722 10.1111/bjh.16384

[6] Zappitelli M, Moffett BS, Hyder A, Goldstein SL. Acute kidney injury in non-critically ill children treated with aminoglycoside antibiotics in a tertiary healthcare centre: a retrospective cohort study. Nephrol Dial Transpl. 2011;26:144–50. 10.1093/ndt/gfq375. 10.1093/ndt/gfq375 20591815

[7] Searns JB, Gist KM, Brinton JT, Pickett K, Todd J, Birkholz M, et al. Impact of acute kidney injury and nephrotoxic exposure on hospital length of stay. Pediatr Nephrol. 2020;35:799–806. 10.1007/s00467-019-04431-3. 31940070 10.1007/s00467-019-04431-3

[8] Misurac JM, Knoderer CA, Leiser JD, Nailescu C, Wilson AC, Andreoli SP. Nonsteroidal anti-inflammatory drugs are an important cause of acute kidney injury in children. J Pediatr. 2013;162:1153–9. 10.1016/j.jpeds.2012.11.069. 23360563 10.1016/j.jpeds.2012.11.069

[9] Steflik HJ, Brinton DL, Corrigan C, Wagner CL, Selewski DT, Twombley KE, et al. Costs associated with acute kidney injury in critically Ill neonates with patent Ductus arteriosus: pediatric health information system (PHIS) analysis. J Perinatol. 2022. 10.1038/s41372-022-01499-y. 10.1038/s41372-022-01548-6 36329163

[10] Feudtner C, Feinstein JA, Zhong W, Hall M, Dai D. Pediatric complex chronic conditions classification system version 2: updated for ICD-10 and complex medical technology dependence and transplantation. BMC Pediatr. 2014;14:199. 10.1186/1471-2431-14-199. 25102958 10.1186/1471-2431-14-199 PMC4134331

[11] Feudtner C, Christakis DA, Connell FA. Pediatric deaths attributable to complex chronic conditions: a population-based study of Washington State, 1980-1997. Pediatrics. 2000;106:205–9. 10888693

[12] International statistical classification of diseases and related health problems. World Health Organization; 2019. https://icd.who.int/. 3376487

[13] Jetton JG, Guillet R, Askenazi DJ, Dill L, Jacobs J, Kent AL, et al. Assessment of Worldwide Acute Kidney Injury Epidemiology in Neonates: Design of a Retrospective Cohort Study. Front Pediatr. 2016;4:68. 10.3389/fped.2016.00068. 27486571 10.3389/fped.2016.00068 PMC4950470

[14] United States Bureau of Labor Statitics. Consuper Price Index (CPI) for all Urban Consumers (CPI-U): U.S. cit average, for inpatient hospital services, not seasonally adjusted [dataset]. Retrieved from: https://www.bls.gov/news.release/cpi.t02.htm. Data accessed: 4 Dec 2024.

[15] Yu W, Xu W, Zhu L. A modified Hosmer-Lemeshow test for large data sets. Commun Stat Theory Methods. 2017;46:11813–25.

[16] Gibbons J, Chakraborti S. Nonparametric Statistical Inference. 5th ed. ed. New York: Chapman & Hall; 2010.

[17] Jonckheere A. A distribution-free k-sample test against ordered alternatives. Biometrika. 1954;41:133–45.

[18] Terpstra T. The asymptotic normality and consistency of Kendall’s test against trend, when ties are present in one ranking. Indagationes Mathematicae. 14:327–33.

[19] von Elm E, Altman DG, Egger M, Pocock SJ, Gøtzsche PC, Vandenbroucke JP. The Strengthening the Reporting of Observational Studies in Epidemiology (STROBE) statement: guidelines for reporting observational studies. Ann Intern Med. 2007;147:573–7. 10.7326/0003-4819-147-8-200710160-00010. 17938396 10.7326/0003-4819-147-8-200710160-00010

[20] Daga A, Dapaah-Siakwan F, Rajbhandari S, Arevalo C, Salvador A. Diagnosis and Risk Factors of Acute Kidney Injury in Very Low Birth Weight Infants. Pediatr Neonatol. 2017;58:258–63. 10.1016/j.pedneo.2016.08.002. 27773638 10.1016/j.pedneo.2016.08.002

[21] Momtaz HE, Sabzehei MK, Rasuli B, Torabian S. The main etiologies of acute kidney injury in the newborns hospitalized in the neonatal intensive care unit. J Clin Neonatol. 2014;3:99–102. 10.4103/2249-4847.134691. 25024976 10.4103/2249-4847.134691 PMC4089136

[22] Majed B, Bateman DA, Uy N, Lin F. Patent ductus arteriosus is associated with acute kidney injury in the preterm infant. Pediatr Nephrol. 2019;34:1129–39. 10.1007/s00467-019-4194-5. 30706125 10.1007/s00467-019-4194-5

[23] Soilly AL, Lejeune C, Quantin C, Bejean S, Gouyon JB. Economic analysis of the costs associated with prematurity from a literature review. Public Health. 2014;128:43–62. 10.1016/j.puhe.2013.09.014. 24360723 10.1016/j.puhe.2013.09.014

[24] Schmitt SK, Sneed L, Phibbs CS. Costs of newborn care in California: a population-based study. Pediatrics. 2006;117:154–60. 10.1542/peds.2005-0484. 16396873 10.1542/peds.2005-0484 PMC8720276

[25] Cheah IGS. Economic assessment of neonatal intensive care. Transl Pediatr. 2019;8:246–56. 10.21037/tp.2019.07.03. 31413958 10.21037/tp.2019.07.03 PMC6675687

[26] Younge N, Goldstein RF, Bann CM, Hintz SR, Patel RM, Smith PB, et al. Survival and Neurodevelopmental Outcomes among Periviable Infants. N Engl J Med. 2017;376:617–28. 10.1056/NEJMoa1605566. 28199816 10.1056/NEJMoa1605566 PMC5456289

[27] Phibbs CS, Schmitt SK. Estimates of the cost and length of stay changes that can be attributed to one-week increases in gestational age for premature infants. Early Hum Dev. 2006;82:85–95. 10.1016/j.earlhumdev.2006.01.001. 16459031 10.1016/j.earlhumdev.2006.01.001 PMC1752207

[28] Russell RB, Green NS, Steiner CA, Meikle S, Howse JL, Poschman K, et al. Cost of hospitalization for preterm and low birth weight infants in the United States. Pediatrics. 2007;120:e1–9. 10.1542/peds.2006-2386. 17606536 10.1542/peds.2006-2386

[29] Geitona M, Hatzikou M. Cost esimtation of neonatal intensive care in Greece: the case of Athens maternity hospitals. J Med Econ. 2007;3:273–83.

[30] May P, Garrido MM, Cassel JB, Morrison RS, Normand C. Using Length of Stay to Control for Unobserved Heterogeneity When Estimating Treatment Effect on Hospital Costs with Observational Data: Issues of Reliability, Robustness, and Usefulness. Health Serv Res. 2016;51:2020–43. 10.1111/1475-6773.12460. 26898638 10.1111/1475-6773.12460 PMC5034210

[31] Johnson TJ, Patel AL, Jegier BJ, Engstrom JL, Meier PP. Cost of morbidities in very low birth weight infants. J Pediatr. 2013;162:243–49.e1. 10.1016/j.jpeds.2012.07.013. 22910099 10.1016/j.jpeds.2012.07.013 PMC3584449

[32] Valencia E, Staffa SJ, Kuntz MT, Zaleski KL, Kaza AK, Maschietto N, et al. Transcatheter Ductal Stents Versus Surgical Systemic-Pulmonary Artery Shunts in Neonates With Congenital Heart Disease With Ductal-Dependent Pulmonary Blood Flow: Trends and Associated Outcomes From the Pediatric Health Information System Database. J Am Heart Assoc. 2023;12:e030528. 10.1161/jaha.123.030528. 37589149 10.1161/JAHA.123.030528 PMC10547312

[33] Keane OA, Zamora AK, Ourshalimian S, Mahdi EM, Song AY, Kim E, et al. Opioid and Methadone Use for Infants With Surgically Treated Necrotizing Enterocolitis. JAMA Netw Open. 2023;6:e2318910. 10.1001/jamanetworkopen.2023.18910. 37347485 10.1001/jamanetworkopen.2023.18910 PMC10288332

[34] Roy JP, Goldstein SL, Schuh MP. Under-Recognition of Neonatal Acute Kidney Injury and Lack of Follow-Up. Am J Perinatol. 2022;39:526–31. 10.1055/s-0040-1716841. 32971562 10.1055/s-0040-1716841

[35] Chmielewski J, Chaudhry PM, Harer MW, Menon S, South AM, Chappell A, et al. Documentation of acute kidney injury at discharge from the neonatal intensive care unit and role of nephrology consultation. J Perinatol. 2022;42:930–6. 10.1038/s41372-022-01424-3. 35676535 10.1038/s41372-022-01424-3 PMC9280854

