# Supplementary material for: Acute kidney injury associated with increased costs in the neonatal intensive care unit: analysis of Pediatric Health Information System database
Source: J Perinatol. 2024 Dec 5;45(1):94–100. doi: 10.1038/s41372-024-02193-x (PMC11711086; doi:10.1038/s41372-024-02193-x)
Supplement: Supplementary file 1 — Suplemental Figure [file 41372_2024_2193_MOESM1_ESM.pdf]

Supplemental Table 1. Feudtner Pediatric Complex Chronic Conditions Classification (CCC) Premature and Neonatal Category and Corresponding International Classification of Disease, Ninth and Tenth Revision, Clinical Modification Diagnosis Codes

Feudtner[†] Pediatric Complex Chronic Conditions (CCC) Classifications Category	Sub-categories	ICD-9	ICD-10
**Premature and Neonatal**	Fetal malnutrition	764.01, 764.02, 764.11, 764.12, 764.21, 764.22, 764.91, 764.92	P05.01, P05.11, P05.02, P05.12, P05.2, P05.9
	Extreme immaturity	765.01, 765.02, 765.11, 765.12, 765.21-765.23	P07.01, P07.02, P07.21-P07.25
	Cerebral hemorrhage at birth	767.0	P10.0, P10.1, P10.4, P52.4, P52.8
	Spinal cord injury at birth	767.4	P11.5
	Birth asphyxia	768.5, 768.9	P21.0, P21.9, P84
	Respiratory diseases	770.2, 770.7	P25.0-P25.3, P25.8, P27.0, P27.1, P27.8
	Hypoxic-ischemic encephalopathy	768.7	P91.6
	Other[†]	771.0, 771.1, 772.13, 772.14, 773.3, 773.4, 774.7, 776.5, 777.53, 778.0, 779.7	P35.0, P35.1, P25.21, P25.22, P56.0, P57.0, P57.8, P61.3, P61.4, P77.3, P83.2, P91.2

Adapted from supplemental materials from Feudtner et al. BMC Pediatrics 2014, 14:199.
[†]Codes comprise infections in the perinatal period, severe intraventricular hemorrhage, hydrops fetalis, kernicterus, congenital anemia, severe necrotizing enterocolitis, conditions involving the integument and temperature regulation of fetus and newborn, interstitial emphysema including pneumomediastinum, and periventricular leukomalacia.

Supplemental Table 2. Least Square Means Estimates of Hospitalization Costs

Parameters		Costs	95 % Confidence Interval	
**Sex**	Male	$2,671	$2,683	$2,658
	Female	*Ref.*	--	--
**Race**	Asian/Pacific Islander/ American Indian	$-2,022	$-2,641	$-1,390
	Black	$-904	$-1,113	$-693
	White	*Ref.*	--	--
	Other	$4,316	$3,970	$4,667
**Gestational Age**	**22-26 weeks'**	**$244,948**	**$240,512**	**$249,461**
	**27-30 weeks'**	**$179,351**	**$176,576**	**$182,168**
	**31-34 weeks**	**$54,354**	**$53,775**	**$54,939**
	35-37 weeks'	$8,830	$8,683	$8,978
	≥38 weeks'	*Ref.*	--	--
**Renal Replacement Therapy**	Yes	$131,981	$115,214	$150,421
**Feudtner[†] Pediatric Complex Chronic Conditions (CCC) Classifications**	**Cardiovascular**	**$60,225**	**$59,381**	**$61,078**
	**Congenital or Genetic**	**$53,432**	**$52,182**	**$54,702**
	**Gastrointestinal**	**$49,255**	**$48,108**	**$50,420**

	Hematologic or Immunologic	$22,514	$20,966	$24,102
	Malignancy	$-20,552	$-21,070	$-20,020
	Metabolic	$15,980	$15,070	$16,904
	Neurologic or Neuromuscular	$22,889	$22,052	$23,740
	Renal	$17,124	$16,262	$18,001
	Respiratory	$43,209	$41,894	$44,548
	**Medical Technology (i.e., device dependency)**	**$33,033**	**$31,954**	**$34,130**
	Transplantation	$14,633	$13,021	$16,293
**Renal Replacement Therapy**	Yes	$131,981	$115,214	$150,421

†Feudtner et al. BMC Pediatrics 2014, 14:199.
The top key drivers of AKI-associated costs are indicated in bold: four Feudtner CCCs (cardiovascular, congenital or genetic, gastrointestinal, medical technology) and gestational age.
All p-values <0.0001.

Supplemental Figure. Length of Hospitalization by Gestational Age Group among Neonates With and Without Acute Kidney Injury

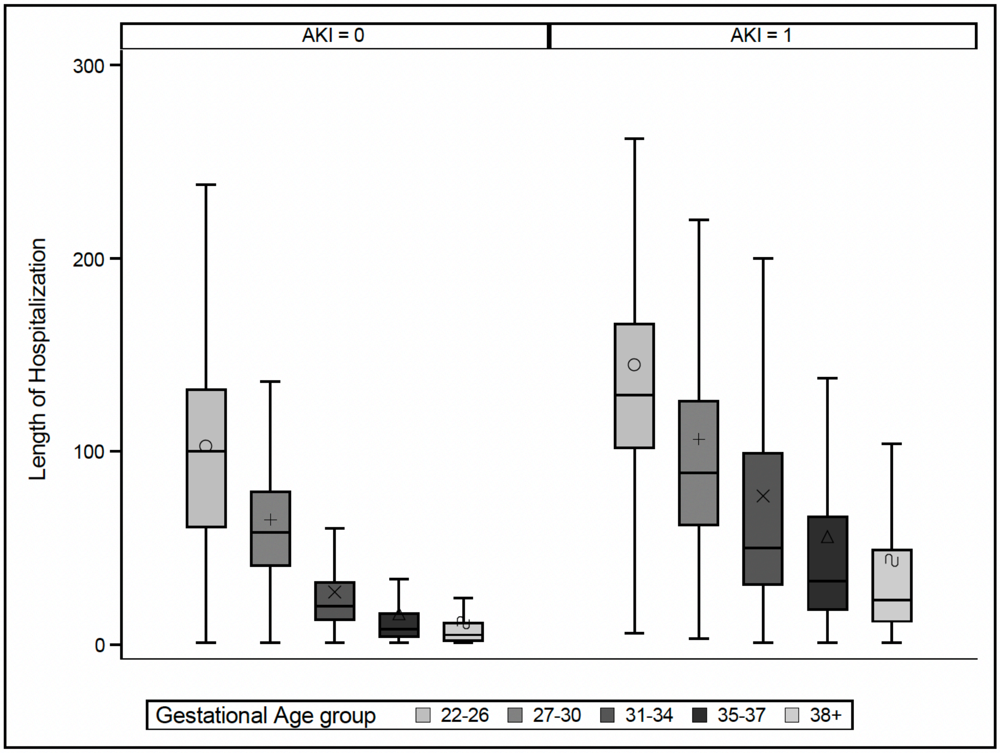

Gestational Age (completed weeks of gestation)	Neonates without AKI (AKI=0)			Neonates with AKI (AKI=1)		
	N (observations)	Length of Hospitalization (days)		N (observations)	Length of Hospitalization (days)	
		Mean	Median[1]		Mean	Median[2]
22-26 weeks	9,991	103.0	100	1,682	144.7	129
27-30 weeks	17,278	64.7	58	669	106.4	89
31-34 weeks	45,878	27.2	20	764	76.9	50
35-37 weeks	60,955	16.0	8	1,433	55.9	33
≥38 weeks	94,963	11.3	5	2,475	43.6	23

Box-and-whiskers plot illustrating length of hospitalization among the various gestational age groups. The Box is defined by the 25th and 75th percentiles. Within the Box, the mean length of hospitalization is indicated by the notation (open circle, cross, X, triangle, curved line, respectively) and the median length of hospitalization is indicated by the horizontal bold bar. The whiskers indicated the minimum and maximum observations.

[1,2] Significant for non-linear trend (p<0.0001) using the Jonckheere-Terpstra test
